# Etude échographique du diamètre de l'enveloppe du nerf optique chez l'enfant noir africain sain

**DOI:** 10.11604/pamj.2014.19.285.4600

**Published:** 2014-11-15

**Authors:** Kofi-Mensa Savi de Tové, Olivier Biaou, Julien Didier Adedemy, Olatoundji Holden Fatigba, Patricia Yèkpè, Vicentia Boco, Augustin Karl Agossou-Voyeme

**Affiliations:** 1 Faculté de Médecine, Université de Parakou, Bénin; 2 Faculté des Sciences de la Santé, Université d'Abomey Calavi, Bénin

**Keywords:** Diamètre de l′enveloppe du nerf optique, échographie, enfant, noir africain, Bénin, Sonographic study of the diameter of the sheat of the optic nerve in healthy black African children, ultrasound, child, black african, Benin

## Abstract

**Introduction:**

L'objectif de cette étude était de déterminer le diamètre échographique de l'enveloppe du nerf optique (DENO) dans une population d'enfants sains noirs Africains au Bénin.

**Méthodes:**

Une étude transversale descriptive a été menée sur une période de 6mois. Le DENO a été mesuré chez 304 enfants sains. Deux mesures échographiques du DENO (coupe transversale et sagittale) ont été réalisées 3mm en arrière de la papille sur chaque œil. Le DENO d'un patient est égal à la moyenne des quatre mesures.

**Résultats:**

L’âge moyen était de 35, 72 ± 35,38 mois et la sex-ratio H/F de 0,96. La mesure moyenne du DENO était de 3, 31±0,54mm avec des extrêmes de 2,02 et de 4,44mm. Le DENO croît avec l’âge avec une moyenne corrélation significative (r = 0,58 et p < 0,0001). Cette croissance est plus marquée pendant les 48 premiers mois de vie. Il n'y avait pas de différence entre les garçons et les filles (p = 0, 45).

**Conclusion:**

Les valeurs retrouvées dans cette étude ne diffèrent pas de ce qui est classiquement décrit dans les autres populations. Un DENO supérieur à 4,40 (IC 95%) doit être considéré comme anormal.

## Introduction

L'hypertension intracrânienne (HIC) est une urgence diagnostique et thérapeutique responsable de la létalité de plusieurs affections neurologiques de l'enfant [1]. Elle est définie comme l’élévation de la pression intracrânienne (PIC) au delà des valeurs physiologiques [2]. La méthode diagnostic de référence est la mesure continue par capteur intra ventriculaire ou intra parenchymateux de la PIC [3]. Cette technique invasive avec de possibles complications infectieuses ou hémorragiques, n'est pas disponible dans notre contexte de travail. Le fond d’œil aide au diagnostic en montrant un œdème papillaire mais, ce signe est inconstant et d'installation tardive [1]. En 1997, Hansen et al. ont démontré que les variations de la PIC induites par l'injection intrathécale de Ringer lactate entraînaient des variations du diamètre de l'enveloppe du nerf optique (DENO) mesuré par échographie [4, 5]. Ceci s'explique par la disposition anatomique de la portion intra orbitaire du nerf optique (NO) qui est en continuité avec les espaces sous arachnoïdiens. De nombreuses études ont montré l'utilité de la mesure du DENO dans le diagnostic des HIC aussi bien chez l'adulte que chez l'enfant [2, 6–11]. Il s'agit d'une technique fiable, non invasive et pouvant être répétée à souhait sans inconvénient [2, 11]. Cette technique diagnostic constitue pour les pays sous médicalisés comme le notre une excellente alternative à la mesure continue de la PIC en cas de suspicion d'HIC. Le but de ce travail était de déterminer par échographie la valeur normale du DENO dans une population d'enfants sains noirs africains.

## Méthodes

Cette étude s'est déroulée à Parakou, ville septentrionale de la République du Bénin. Parakou est situé à environ 400 km de Cotonou la capitale. Le Centre Hospitalier départemental du Borgou est un hôpital de référence de 2ème degré sur la pyramide sanitaire du Bénin. Les services de pédiatrie et d'imagerie médicale de cet hôpital ont servi de cadre à cette étude. Il s'est agi d'une étude transversale descriptive et analytique avec recueil prospectif des données menée sur une période de 6 mois allant du 01 mars au 30 août 2013.

**Figure 1:**
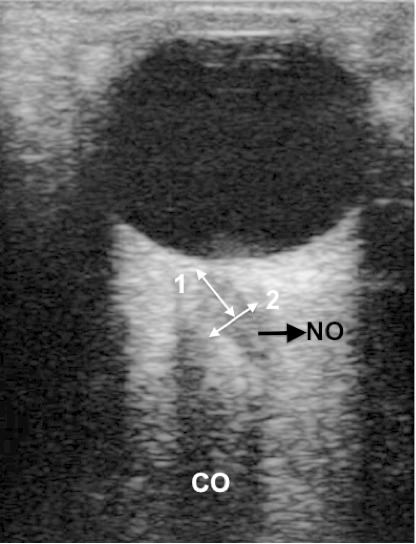
Image échographique du nerf optique montrant la technique de mesure du nerf optique (NO). Le NO est mesuré trois millimètres en arrière en arrière de la papille (1), perpendiculairement à son axe les curseurs étant positionnés sur les limites externes des gaines (2). On visualise également l'artéfact à type de cône d'ombre (CO) qu'il faut prendre soin de différencier du NO

L’échantillon est constitué de 304 enfants sains âgés de 15ans au plus. Ils ont été recrutés parmi des enfants reçus sur rendez-vous en consultation pédiatrique durant la période d’étude. Il s'agissait d'enfants vus pour le contrôle d'une affection non neurologique et qui ont été déclarés guéris. Tous les enfants ont été examinés par un médecin neurologue afin d’éliminer tout trouble neurologique, en particulier ceux évoquant une HIC. Tous les enfants présentant une pathologie orbito oculaire empêchant la réalisant de l’échographie du NO n'ont pas été retenus. Les échographies du NO ont été réalisées par un médecin radiologue, grâce à un appareil d’échographie mode B muni d'une sonde linéaire multi fréquence (5-10MHz). Les échographies ont été réalisés sans prémédication, enfants en décubitus dorsal. Nous avons surtout pour les petits enfants sollicité l'aide des parents pour stabiliser leur tête durant l'examen. Après application d'une fine couche de gel sur la paupière supérieure close, la sonde était placée sur cette dernière en position médiane ou temporale en prenant soin d’éviter toute pression excessive sur le globe oculaire. Chez les grands enfants, nous leur demandions de diriger le regard au zénith afin de positionner la papille et le NO en regard de la sonde (Figure 1). Chez les enfants plus petits et ceux chez qui une coopération n'a pu être obtenue, la sonde d’échographie était manipulée jusqu’à l'obtention d'une image satisfaisante du NO. Dans ces cas le mode « loop » ou « mode cinéma » a été d'une grande utilité. Le disque optique se présente sous la forme d'une hyperéchogénicité linéaire située au pôle postérieur du globe oculaire. Le NO au sein de la graisse rétrobulbaire hyperéchogène est individualisé comme une colonne hypoéchogène entourée par une gaine hyperéchogène (Figure 1).

Chez chaque enfants, deux coupes échographiques sont réalisées; une coupe transversale, la sonde étant positionnée horizontalement et une coupe sagittale, la sonde positionnée verticalement. Sur chacune de ces coupes le DENO est mesuré 3mm en arrière de la papille et perpendiculairement à l'axe du NO, le curseur électronique étant positionné sur les limites externes de l'enveloppe (Figure 1). Ainsi, nous avons réalisé quatre mesures chez chaque enfant. La valeur du DENO d'un patient est égale à la moyenne de ces quatre mesures [9, 12].

DENO=(DVOD + DHOD + DVOG + DHOG)/4

DVOD = DENO Vertical œil Droit DHOD = DENO Horizontal œil Droit

DVOG = DENO Vertical œil Gauche DHOD = DENO Horizontal œil Gauche

Les différentes variables étudiées ont été l’âge, le sexe et le diamètre échographique de l'enveloppe du NO. Les données ont été traitées grâce aux logiciels Epi info version 3.5.1. et XLSTAT version 2013. Les corrélations ont été appréciées grâce au coefficient de corrélation r de Bravais pearson. Cette étude a obtenu l'aval du comité local d’éthique et le consentement verbal éclairé (dans la langue autochtone) des parents de tous les enfants inclus dans l’étude.

## Résultats

**Figure 2:**
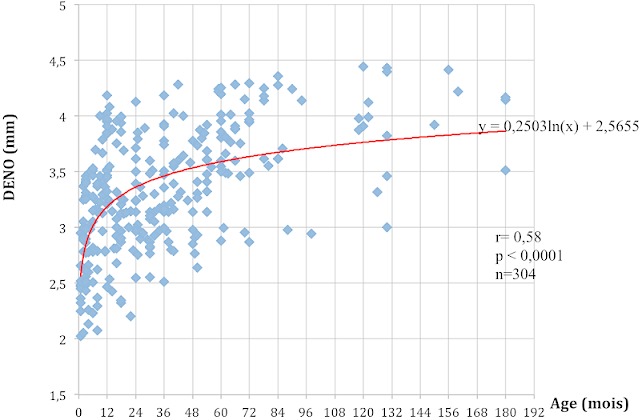
Courbe de régression logarithmique montrant l’évolution du DENO (en mm) en fonction de l’âge (en mois)

La mesure du DENO a pu être réalisée chez tous les 304 enfants de notre échantillon. Il y avait 149 garçons (49,01%) et 155 filles (50,99%) soit une sex-ratio H/F de 0,96. L’âge moyen était de 35,72 ± 35,38 mois avec des extrêmes de 1 et 180 mois (15ans). Dans notre série, la valeur moyenne du DENO était de 3,31±0,54 mm avec des extrêmes de 2,02 et de 4,44mm. Elle était de 3,31 ± 0,54 mm à droite et de 3,30 ± 0,53mm et à gauche. La différence moyenne entre les deux yeux était de 0,01355 ± 0,08450 avec des extrêmes de 0,023 et de 0,004mm. Le DENO moyen en coupe transversal était de 3,31±0,54mm et de 3,30±0,53mm en coupe sagittale. La différence moyenne entre les deux coupes 0,014±0,80 avec des extrêmes de 0,005 et 0,023mm. Le Tableau 1 résume les valeurs du DENO en fonction du sexe et la Figure 2 montre l’évolution du DENO en fonction de l’âge. Le DENO croît avec l’âge avec une moyenne corrélation significative (r = 0,58 et p < 0,0001). Cette croissance du DENO est plus marquée pendant les 48 premiers mois de vie. Le rythme de croissance diminue ensuite progressivement jusqu’à 156 mois (13ans). Le Tableau 2 compare les valeurs du DENO chez les enfants de moins de 48 mois et ceux plus âgés.

**Tableau 1:** Valeurs du DENO en millimètre en fonction du sexe

	n (%)	Moy ± ET	min	max
Garçons	149 (49,01)	3,28±0,58	2,02	4,43
Filles	155 (50,99)	3,34±0,50	2,23	4,44
Total	304 (100)	3,31±0,54	2,02	4,44

CHI2 = 0,5516; p = 0,4577

N= effectif Moy= moyenne ET= écart type min= minimum max = maximum

**Tableau 2:** Valeurs du DENO en mm chez les enfants de moins de 48 mois et ceux plus âgés

	n (%)	moy ± ET	min	max
< 48 mois	213(70)	3,14±0,49	2,02	4,28
≥ 48 mois	91(30)	3,70±0,45	2,64	4,44
Total	304(100)	3,30±0,53	2,02	4,44

CHI2 = 66,9727; p = 0,0001

N= effectif Moy= moyenne ET= écart type min= minimum max = maximum

## Discussion

Cette étude nous a permis de déterminer la valeur moyenne et les limites du DENO chez l'enfant noir africain sain. Le DENO moyen dans cette étude (3.31±0.55mm) se rapproche de celui retrouvé dans d'autres populations [4, 6, 8, 13, 14] (Tableau 3). La mesure échographique du DENO est une technique non invasive, fiable et reproductible de détection d'une HIC. Plusieurs études ont montré que les différences inter et intra observateur étaient négligeables lors de cette mensuration [9, 15–20]. La technique de mesure du DENO est variable selon les études mais l'unanimité est faite sur le fait qu'il doit être mesuré 3mm en arrière de la papille, zone du nerf optique la plus sensible aux variations de la pression intracrânienne [4, 5]. En ce qui concerne la position du regard lors de cette mesure, Roberto Copetti et al ont souligné le risque de surestimer les mesures réelles du DENO en mesurant un artéfact (probablement projeté par la lamina cribosa) à la place du NO lorsque le regard du sujet est en position médiane [21] (Figure 1). Ils proposent donc de mesurer le DENO avec le regard du sujet en position latérale. Dans notre étude, nous avons mesuré le nerf optique sans tenir compte de la position du regard mais en prenant soin de bien différencier le nerf optique de l'artefact, par une bonne visualisation de la gaine hyperéchogène du NO (Figure 1).

**Tableau 3:** Valeurs moyennes du nerf optique des enfants sains selon différentes études

Auteurs	Année	Pays	Effectif	Moyenne
Helmke et al. [4]	1996	Allemagne	102	3.10 mm
Ballantyne et al. [6]	1999	Royaume Unis	102	3,08 ± 0,36 mm
Malayeri et al. [8]	2005	Iran	78	3,30 ± 0,60 mm
Körber et al. [13]	2005	Allemagne	466	3,40 ± 0,7 mm
Beare et al [14]	2008	Malawi	30	3,5 mm
Notre étude	2013	Bénin	304	3,31 ± 0,55 mm

Les plans de mesure du DENO utilisés lors des différentes études sont également variables. Plusieurs auteurs ont utilisés exclusivement les mesures dans le plan axial [8, 10, 22]. Certains auteurs ont remarqué que les mesures du DENO sur ces coupes axiales étaient significativement inférieures à celles réalisées sur des coupes sagittales [4]. Pour plusieurs auteurs tout comme nous utilisé la moyenne des mesures dans les plans axial et sagittal pour déterminer le DENO constitue le meilleur compromis [12, 15, 17, 23, 24]. Le DENO ne varie pas en fonction du sexe dans notre série (Tableau 1). Plusieurs auteurs dont Ballantyne [6] et Maude [23] avaient déjà souligné ce fait. Dans notre échantillon le DENO augmente avec l’âge (r = 0.58). Cette croissance du DENO se faisant surtout au cours des 48 premiers mois de vie (Figure 2) avec une différence significative entre le DENO des enfants de moins de 48 mois et ceux plus âgés (p = 0,0001) (Tableau 2). Ce constat rejoint celui fait par plusieurs auteurs dont Helmke et al. en Allemagne et Malayari en Iran [8] qui ont également montré dans leur série que la croissance du DENO se produit surtout durant les 04 premières années de vie [4]. Au delà de cet âge, la valeur du DENO se rapproche de celle observée à l’âge adulte [23].

## Conclusion

Au terme de cette étude on peut considérer dans nos populations qu'un DENO supérieur à 4 mm chez les enfants de moins de 48 mois, et à 4,40 mm chez ceux plus âgés devrait être considéré comme augmenté, suggérant une HIC.
